# Identification of key microRNAs and their targets in exosomes of pancreatic cancer using bioinformatics analysis

**DOI:** 10.1097/MD.0000000000012632

**Published:** 2018-09-28

**Authors:** Xin Zhao, Yiming Ren, Naiqiang Cui, Ximo Wang, Yunfeng Cui

**Affiliations:** a Tianjin Medical University, Tianjin; b Department of Surgery, Tianjin Nankai Hospital, Nankai Clinical School, Tianjin Medical University; c Department of Bone and Joint, Tianjin Union Medicine Center, PR China.

**Keywords:** bioinformatics analysis, microRNAs, pancreatic cancer, serum exosomes, target genes

## Abstract

Pancreatic cancer (PC) is one of the most lethal tumors, due to late diagnosis and limited surgical strategies. It has been reported that serum exosomal microRNAs (S-Exo-miRNAs) play a pivotal role as signaling molecules and serve as noninvasive diagnosis methods for PC. The combination of S-Exo-miRNAs with the corresponding target also plays an important role in the tumor microenvironment.

Here we investigated S-Exo-miRNAs involved in PC. The gene expression profile was downloaded from the Gene Expression Omnibus (GEO) database. The analysis was carried out using GEO2R. The targets of differentially expressed serum exosomal miRNAs (DE-S-Exo-miRNAs) were predicted by 4 bioinformatic algorithms (miRanda, miRDB, miRWalk, and Targetscan). Further analysis with gene ontology (GO) and Kyoto Encyclopedia of Genomes pathway (KEGG) enrichment analyses were performed with Cytoscape software version 3.4.0. Subsequently, the interaction regulatory network of target genes was performed with the Search Tool for the Retrieval of Interacting Genes (STRING) database (http://www.string-db.org/) and visualized using Cytoscape software.

We downloaded the gene expression profile GSE50632, which was based on an Agilent microarray GPL17660 platform containing 4 eligible samples. In total 467 DE-S-Exo-miRNAs were obtained, including 7 overexpressed miRNAs (1.50%), and 460 remaining underexpressed miRNAs (98.50%). The databases miRWalk, miRDB, miRanda, and TargetScan were used to predict their potential targets, which were subsequently submitted to Cytoscape software version 3.4.0 (www.cytoscape.org). Next the functional and pathway enrichment analysis were used for the KEGG pathway and GO categories analysis. The enrichment analysis identified the genes involved in such processes as developmental and negative regulation of multicellular organismal processes, regulation of anatomical structure morphogenesis, regulation of cell death, apoptotic processes and mitogen-activated protein kinase (MAPK) signaling pathway, transforming growth factor - beta (TGF -β) signaling pathway, cyclic adenosine monophosphate (cAMP) signaling pathway, and the phosphatidylinositol-3 kinases/Akt (PI3K-Akt) signaling pathway. Subsequently according to the protein–protein interaction (PPI) network, the top 10 genes were obtained. The enrichment analyses of the genes involved in a significant module revealed that these genes were related to the TGF-β signaling pathway. After reviewing the literature, we identified the apoptosis genes, and their corresponding miRNAs that have a relationship with apoptosis of the tumor.

This analysis provides a comprehensive understanding of the roles of S-Exo-miRNAs and the related targets in the development of PC. Additionally, the present study provides promising candidate targets for early diagnosis and therapeutic intervention. However, these predictions require further experimental validation in future studies.

## Abstract 2

Supplemental Digital Content is available in the text

## 1 Introduction

Pancreatic cancer (PC) is one of the most lethal types of malignancies, with an average 5-year survival rate of less than 5%.^[1]^ One of the latest cancer statistics in 2016 indicated that PC caused approximately 227,000 deaths per year worldwide.^[2]^ Due to the asymptomatic onset of PC, as well as the lack of improvement in early detection and desirable treatment strategies, disease often appears after the tumor invades the surrounding tissues and patients already have late-stage cancer.^[3,4]^ Therefore, understanding the molecular mechanism and finding novel biomarkers to detect PC is urgently required. Considering a noninvasive/minimally invasive and sensitive diagnostic method is a heavily researched topic in research at present and studies on the serum markers have made some progress.

MicroRNAs (miRNA) are a class of endogenous, noncoding RNAs (19–22 nucleotide) that regulate post-transcriptional gene expression by binding to complementary seed sequences at the untranslated regions (UTRs) of their target mRNAs.^[5]^ There was evidence that miRNAs are involved in many pathological cancer processes.^[6,7]^ In 2007, Valadi et al^[8]^ firstly extracted miRNAs in exosomes. These researchers confirmed that exocrine secreted by mast cells could transport miRNAs, mRNAs, and other substances to target cells. Since the publication of that research, the number of studies examining miRNAs in exosomes has been increasing. Correspondingly, studies have investigated that exosomal miRNA is a novel serum biomarker for the early diagnosis of PC and may play key roles in biological processes such as cell proliferation, apoptosis and differentiation.^[9,10]^

Exosomes (Exo) are small 40 to 100-nm vesicles that primarily contribute to intercellular communication and are derived from the fusion of the intraluminal vesicles of multivesicular bodies (MVB) with the plasma membrane. Exosomes contain selected proteins, mRNAs, and miRNAs, which are secreted from living cells, which could be found in the human body fluids; these exosomes participate communication between the cells.^[11,12]^ Unlike circulating miRNAs, exosomes are enriched in the circulatory system and are protected from RNase degradation which can be more easily detected in the body fluid circulation and microenvironment.^[13]^ The expression profile of miRNAs in exosomes is not the same as the corresponding intracellular miRNAs, indicating that the differentiation of miRNAs is not random but rather has a strict sorting mechanism.^[14]^ Moreover, the expression of serum exosomal microRNAs (S-Exo-miRNAs) may be a better way as they are more informative during tumor carcinogenesis, differentiation and progression. The identification of S-Exo-miRNAs in PC patients demonstrated their potential application in clinical diagnosis or prognosis for PC. It was also concluded that examination of S-Exo-miRNA is a useful serum biomarker for PC diagnosis, aside from serum miRNA.^[15]^ Thus a better understanding of miRNA profiles and their targets in the molecular carcinogenesis of PC would be useful for the early diagnosis and for the therapeutic strategies of PC.

In the present study, we downloaded the gene expression profile GSE50632 from the Gene Expression Omnibus (GEO) database to identify the miRNA profiles in the exosomes of pancreatic patients versus healthy donors. We obtained differentially expressed serum exosomal miRNAs (DE-S-Exo-miRNAs). Subsequently, the target genes of DE-S-Exo-miRNAs were predicted, and performed gene ontology (GO) and Kyoto Encyclopedia of Genes and Genomes (KEGG) enrichment pathway analysis was performed. Finally, the protein–protein interaction (PPI) networks and apoptosis regulatory networks were constructed to investigate the S-Exo-miRNAs involved in the carcinogenesis and progression of pancreatic carcinoma and their related functions for early-stage detection, as well as the therapeutic targets for PC.

## 2 Materials and methods

Ethical approval or patient consent was not required since the present study was a review of previous published literatures.

### 2.1 MiRNA expression microarray data

In the present study, the GSE50632 microarray data was downloaded from the Gene Expression Omnibus (http://www.ncbi.nlm.nih.gov/geo/) database based on the Agilent microarray GPL17660 platform (Agilent—031181 Unrestricted _ Human _ miRNA _ V16.0 _ Microarray 030840), which was submitted by Zoller.^[16]^ Based on the GPL17660 platform, the GSE50632 dataset contained eight samples, and 4 eligible samples, including 2 patient exosome and 2 healthy exosome samples, were selected for our analysis.

### 2.2 DE-S-Exo-miRNAs in patients with or without pancreatic adenocarcinoma

The raw data files used for the analysis included TXT files (Agilent microarray platform). The analysis was carried out using GEO2R, which can perform comparisons on original submitter-supplied processed data tables using the GEO query and limma R packages from the Bioconductor project. The adjusted *P < *.05 and log fold change (FC) >2.0 or log FC < −2.0 were used as the cut-off criteria. The DE-S-Exo-miRNAs between the PC serum and healthy donors’ samples were selected.

### 2.3 Identification of DE-S-Exo-miRNA Target Genes

The genes targeted by DE-S-Exo-miRNAs were predicted by 4 bioinformatic algorithms (miRanda, miRDB, miRWalk, and Targetscan). To improve the accuracy of target gene prediction and reduce the rate of false positives, we selected a common intersection among the 4 databases as a filtering condition. Only the miRNAs that could predict the targets within the 4 databases were further analyzed. Finally, according to the number of targets and the tumor research literature review,^[17–25]^ we observed 9 potential miRNAs for PC research.

### 2.4 GO and KEGG analysis of target genes of DE-Exo-miRNAs

Target genes lists were submitted to the online Cytoscape software version 3.4.0 (www.cytoscape.org) to identify over represented GO categories and pathway categories. GO analysis was used to predict the potential functions of the target gene products in the biological process (BP).^[26]^ The Kyoto Encyclopedia of Genes and Genomes (KEGG, http://www.genome.jp/) is a knowledge base for the systematic analysis of gene functions, linking genomic information with higher level systemic functions.^[27]^

### 2.5 Integration of PPI network and module analysis

Search Tool for the Retrieval of Interacting Genes (STRING) database (http://www.string-db.org/) is an online tool designed to evaluate the PPI information. We submitted 363 target genes predicted by 9 major miRNAs in STRING and only experimentally validated interactions with a combined score >0.4 were selected as significant. Subsequently, the PPI networks were analyzed using Cytoscape software. Then, the plug-in Molecular Complex Detection (MCODE) was applied to screen the modules of the PPI network in Cytoscape. The criteria were set as follows: MCODE score > 3 and the number of nodes > 4. Additionally, the enrichment pathway analysis was performed for genes in the modules. *P < *.05 was considered to be significantly different.

### 2.6 Regulatory network construction

Many of the DE-S-Exo-miRNA target genes we obtained may be PC-associated genes, and it is suggested that the miRNAs that target them may participate in PC progression. Therefore, the regulatory network of the 9 main DE-S-Exo-miRNAs that targeted them were visualized using Cytoscape software, version 3.4.0 (www.cytoscape.org).^[28]^

## 3 Results

### 3.1 Identification of DE-S-Exo-miRNAs

The S-Exo-miRNA profile of GSE50632 was downloaded from the GEO (www.ncbi.nlm.nih.gov/geo/), and the GEO2R method was used to identify DE-Exo-miRNAs in the PC samples compared with the control samples. A *P*-value < .05, adjusted *P*-value < .05, log FC >2.0 or log FC < −2.0 were used as the cut-off criteria. In total, 467 DE-S-Exo-miRNAs were identified including 7 upregulated DE-S-Exo-miRNAs (1.50%) and 460 downregulated DE-S-Exo-miRNAs (98.50%) screened in the PC samples compared with the control samples. The top 50 DE-S-Exo-miRNAs were listed in Table 1. All of the 467 DE-S-Exo-miRNAs are listed in Supplementary Material 1.

**Table 1:**
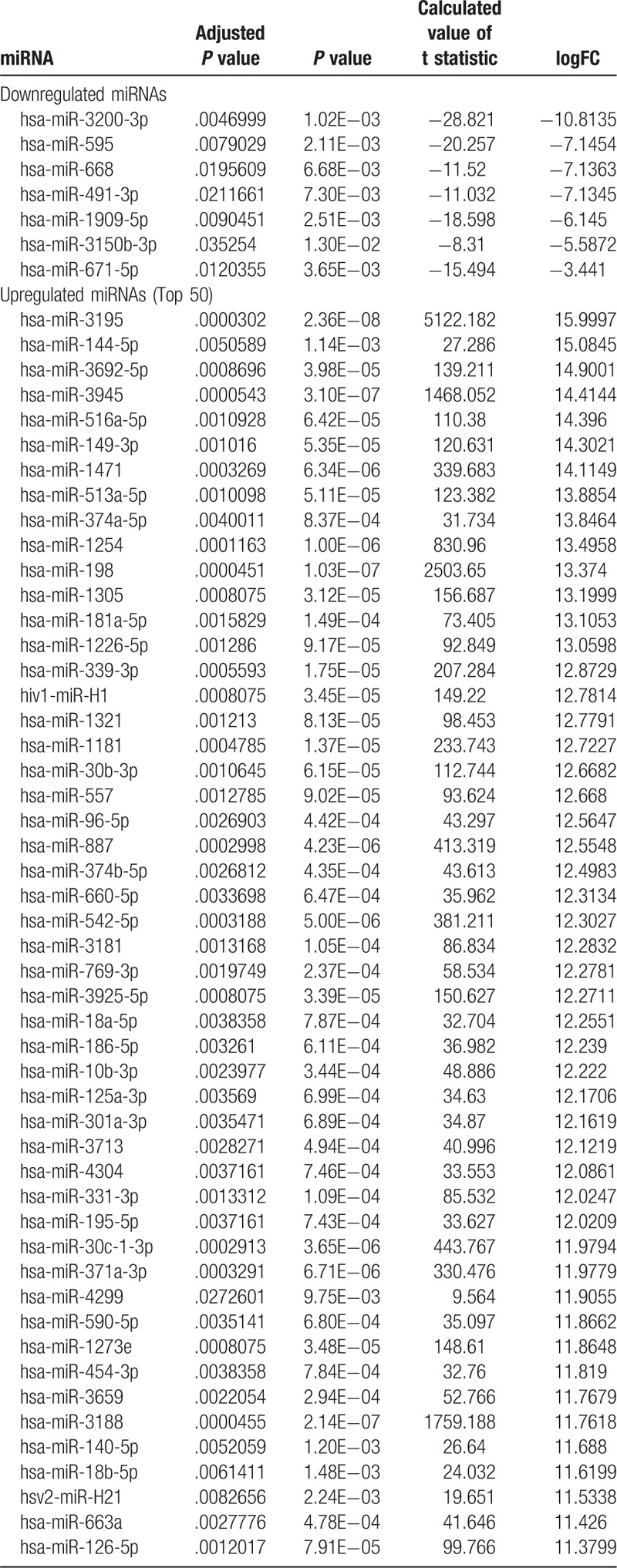
List of differentially expressed exosomal miRNAs.

### 3.2 Prediction of DE-S-Exo-miRNA target genes

To elucidate the functionality of the regulated DE-S-Exo-miRNAs, miRNA gene target prediction was performed for 467 DE-S-Exo-miRNAs using the miRWalk, miRanda, miRDB, and TargetScan databases. To improve the accuracy of the target gene prediction and reduce the rate of false positives, we selected a common intersection as a target gene. Only the miRNAs that could predict the targets with the 4 databases were selected for further analysis. Finally, 26,426 predicted target genes of the 4 upregulated and 146 downregulated DE-S-Exo-miRNAs were obtained.

### 3.3 GO term enrichment and KEGG pathways analysis of target genes of DE-S-Exo-miRNAs

Functional and pathway annotation of the 26,426 target genes were clarified using the Cytoscape software online tool. GO analysis indicated that the target genes were significantly enriched in the negative regulation of developmental process, negative regulation of the multicellular organismal process, regulation of the anatomical structure morphogenesis, regulation of cell death, apoptotic processes, endomembrane system organization, intracellular transport, cell–cell signaling, homeostatic processes, positive regulation of the biological processes, negative regulation of biological process, regulation of metabolic process, regulation of signaling, tube development, nervous system development, and other biological processes (Table 2).

**Table 2:**
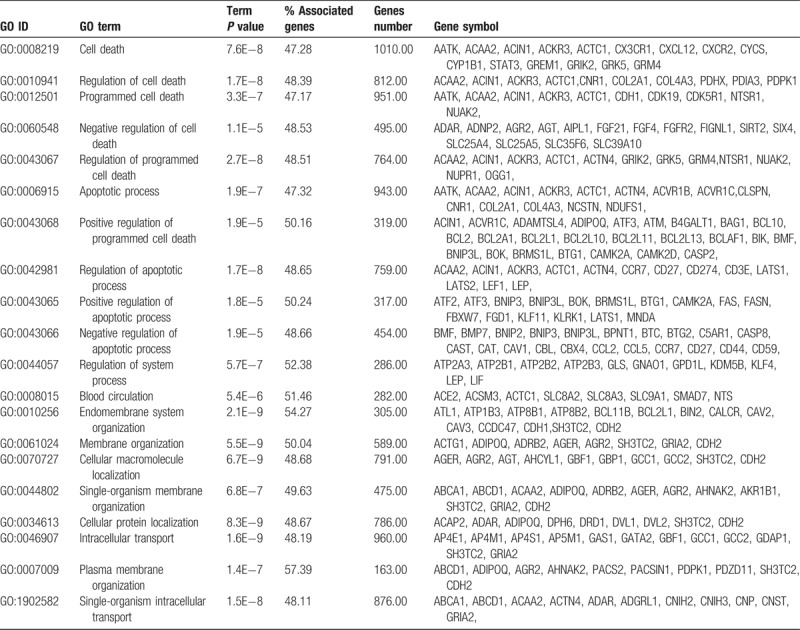
Go function annotation of differentially expression exosomal miRNA target genes. (Top 20).

**Figure 1:**
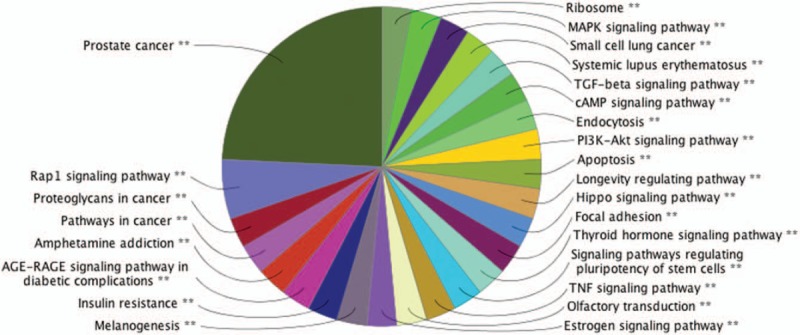
KEGG pathway functional annotation of the differentially expressed exosomal miRNA predicted targets.

The results of KEGG analysis revealed that the target genes were enriched in cancer, ribosome, endocytosis, apoptosis, mitogen-activated protein kinase (MAPK) signaling pathway, transforming growth factor-β (TGF-β) signaling pathways, the cyclic adenosine monophosphate (cAMP) signaling pathway, the phosphatidylinositol-3 kinases/Akt (PI3K-Akt) signaling pathway, hippo signaling pathway, focal adhesion, thyroid hormone signaling pathways, signaling pathways regulating pluripotency of stem cells, the tumor necrosis factor (TNF) signaling pathway, estrogen signaling pathway, insulin resistance, advanced glycation endproducts-receptor for advanced glycation endproducts (AGE-RAGE) signaling pathway, proteoglycans in cancer and Rap1 signaling pathway. These pathways are summarized in Figure 1.

### 3.4 Module screening from the PPI network

**Figure 2:**
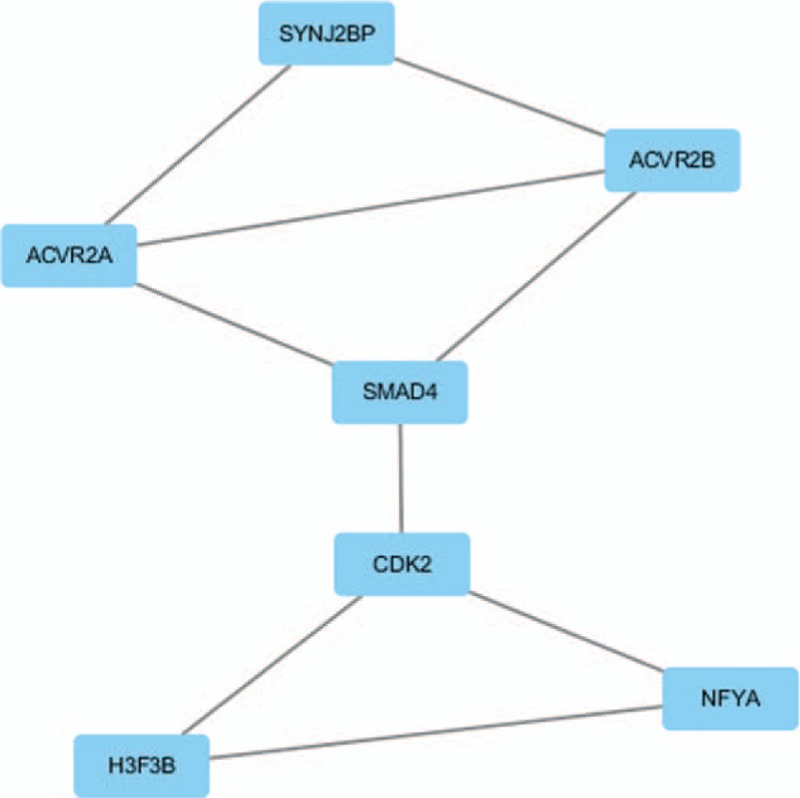
The module from protein–protein interaction network.

According to the number of targets and the review the literature for tumor research, we observed 9 potential miRNAs (hsa-miR-595, hsa-miR-671-5p, hsa-miR-125a-3p, hsa-miR-331-3p, hsa-miR-140-5p, hsa-miR-1225-5p, hsa-miR-490-5p, hsa-miR-630 and hsa-miR-320b) and their corresponding 363 target genes for further research in PC. The PPI network of the 363 target genes predicted by 9 major DE-S-Exo-miRNAs was based on the information in the STRING database. The top 10 nodes with higher degrees were screened. The hub genes including SMAD family member 4 (SMAD4), actin related protein 2 homolog (ACTR2), protein kinase C alpha (PRKCA), SH3-domain GRB2-like 2 (SH3GL2), trans-Golgi network protein 2 (TGOLN2), cyclin-dependent kinase 2 (CDK2), MRT4 homolog (MRTO4), glutamate ionotropic receptor AMPA type subunit 2 (GRIA2), AP2 associated kinase 1 (AAK1) and adenylate cyclase 9 (ADCY9). Moreover, these genes were analyzed using the plug-in MCODE to screen the modules of the PPI network (Fig. 2). The pathway enrichment analysis of the genes involved in a significant module was also performed. The results showed that the genes in a module were primarily associated with the TGF -β signaling pathway.

### 3.5 The regulatory network of the 9 primary DE-S-Exo-miRNAs and their targets

**Figure 3:**
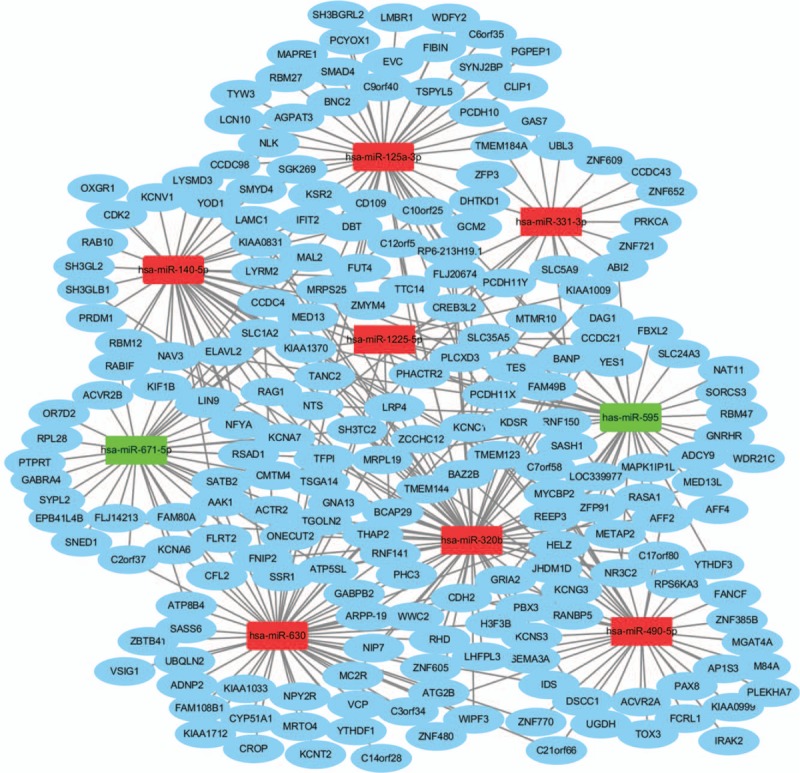
Regulatory network of pancreatic cancer-associated genes and their target miRNAs. (Circle nodes indicate PC-associated genes. Square nodes represent differentially expressed exosomal miRNAs. Red nodes stand for upregulated miRNAs and green nodes for downregulated miRNAs. PC, Pancreatic cancer miRNA, microRNA).

The miRNA-target genes regulatory network was constructed with the miRNA-target gene pairs using the Cytoscape software. With the 363 miRNA-target gene pairs, an miRNA-target gene regulatory network was constructed. In this network, hsa-miR-595, hsa-miR-671-5p, hsa-miR-125a-3p, hsa-miR-331-3p, hsa-miR-140-5p, hsa-miR-1225-5p, hsa-miR-490-5p, hsa-miR-630 and hsa-miR-320b regulate 38, 36, 47, 20, 45, 13, 39, 56, and 69 targets, respectively. Among their targets, the SH3 domain and tetratricopeptide repeats 2 (SH3TC2), GRIA2, phosphatase and actin regulator 2 (PHACTR2), neurotensin (NTS), and N-cadherin (CDH2) were the genes with the highest connectivity (Fig. 3).

### 3.6 Apoptosis associated miRNAs and mRNAs

**Figure 4:**
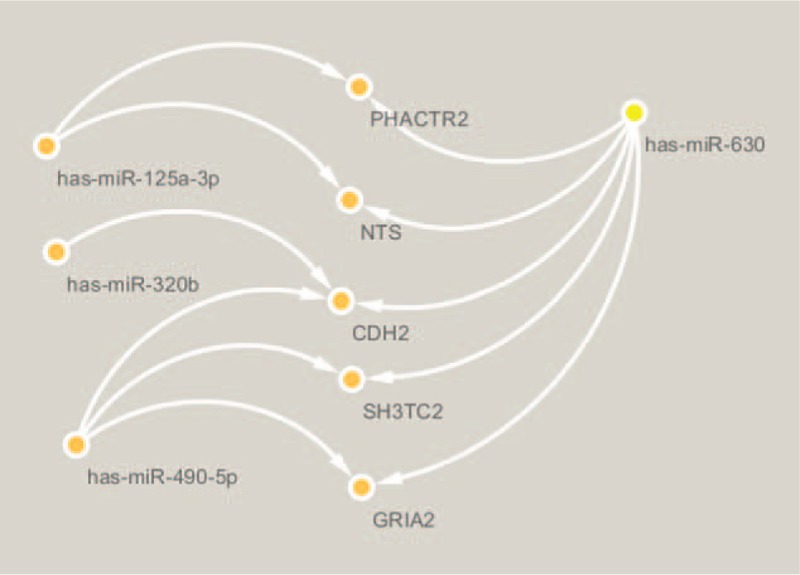
Network of apoptosis associated genes and their target miRNAs.

After searching the database to review the literature about the tumor process according to the results of GO functional and pathway annotation of DE-S-Exo-miRNA target genes, 3 apoptosis genes (NTS, CDH2 and GRIA2) were identified. These apoptosis genes were anticorrelated to the expressions of the DE-S-Exo-miRNA (miR-630, miR-125a-3p, miR-490-5p and miR-320b) (Fig. 4).

## 4 Discussion

In the clinical presentation of PC, it is often insidious and without specific onset symptoms. Thus, early detection of PC remains a challenge for clinical scientists. In the present study, the S-Exo-miRNA expression profile of GSE50632 was downloaded and a bioinformatic analysis was performed. The results showed that there were 467 DE-S-Exo-miRNAs in the serum samples derived from patients with PC and healthy donors. Previous study has termed exosomes that contain both mRNA and microRNA as “exosomal shuttle RNA” (esRNA), which can be delivered to another cell and regulate the target expression in the recipient cell.^[8]^ Subsequent studies have demonstrated that S-Exo-miRNAs are involved in cancer invasion and metastasis by suppressing targeted mRNA in the recipient cells.^[29,30]^ The target relationship between the miRNAs and mRNAs was predicted by the miRWalk, miRanda, miRDB, and TargetScan databases.

According to the number of targets, the results of the function and pathway enrichment analysis and the literature review for tumor research finally identified 9 potential DE-S-Exo-miRNAs and their targets for further analysis. Furthermore, the PPI analysis and the miRNA-targets network of the 9 main DE-S-Exo-miRNAs were performed for a more comprehensive understanding of the molecular mechanisms of PC tumorigenesis.

Regarding the results of GO function and KEGG pathway enrichment analysis, a previous study demonstrated that in the PC process, the tumor microenvironment often exhibits alterations in growth inhibitory pathways and evades apoptosis through p53 mutations and aberrant expression of apoptosis-regulating genes, such as members of the Bcl family, which were consistent with prior results.^[31]^ The apoptosis associated tyrosine kinase (AATK) was induced during growth arrest, could promotes neuronal differentiation of myeloid cells.^[32]^ The study has proposed a hypothesis that the Hippo-YAP pathway is involved in the carcinogenesis of pancreatic cancer and in the inhibition of stiehopus japonieus acidic mucopolysaccharide (SJAMP) in the proliferation of pancreatic cancer cells.^[33]^ This result was consistent with the enrichment function of the positive regulation of apoptotic process. A literature review of the function of cancer-derived exosomes revealed that exosome application decreased the membrane E-cadherin expression, which is important in cell-to-cell adhesion, suggesting a pathway for increased motility and invasiveness in the cancer process.^[34]^ Similarly, a study demonstrated a decrease in cellular E-cadherin expression with the application of cancer-derived exosomes in bladder cancer.^[35]^ In the colon carcinoma cell lines HCT116 and HT29 and pancreatic adenocarcinoma cell line Panc-1, studies have demonstrated that NTS could activate the phosphorylation of both extracellular signal-regulated kinase (ERK) and Akt, in Panc-1 cells through different signaling pathways. NTS stimulated the phosphorylation of ERK and therefore led to epidermal growth factor receptor (EGFR) activation, which may have a closer relationship with the epithelial mesenchymal transition (EMT) and other cancer processes.^[36]^ Studies have shown that NTS stimulates interleukin-8 (IL-8) expression in nontransformed human colonic epithelial cells NCM460 via both the MAPK and nuclear factor-kappaB (NF-κB) dependent pathways.^[37]^ In vivo study of the frog pituitary indicated that NTS could stimulate calcium to affect the inositol triphosphate/protein kinase C pathway.^[38]^ Via suppression of the PI3K/AKT/mTOR signaling pathway, miR-125a-3p inhibits the migration and invasion of cancer cells.^[39]^ This finding was consistent with our KEGG analysis results. Therefore, NTS was inhibited by PC related-exosomal-miR-125a-3p, miR-595 and miR-630, which may be an important mechanism of PC. CDH is expressed normally in mesenchymal cells; however, it has a high expression in various tumors, such as gastric cancer, breast tumors, and nasopharyngeal carcinomas (NPC), inducing cancer cells that become more invasive and motile through the suppression of the Akt3 signaling pathway.^[40]^ Therefore, a comprehensive understanding of the pathways can help us to elucidate the crucial mechanism of PC.

In human glioblastoma tissues, researchers found that miR-595 expression was significantly upregulated could promote cell proliferation by targeting SOX7.^[17]^ Previous study has identified the low expression levels of miR-490-5p in bladder cancer by deep sequencing.^[18]^ Additionally, Zhao et al^[19]^ found that downregulation of miR-630 significantly inhibited cell proliferation, migration and invasion. Reportedly, studies have demonstrated that miR-125a-3p showed a lower expression profile in the patient's serum with pancreatic cancers compared with healthy individuals.^[20,41]^ However, circulating exosomal miR-125a-3p was significantly upregulated in plasma exosomes of patients with early stage colon cancer.^[42]^ Interestingly, the exosomes miR-671-5p has also been shown to be downregulated in serum samples and has the diagnostic capabilities as a biomarker candidate in Kawasaki disease.^[21]^ HCC miRNA array data indicated that miR-331-3p expression in HCC cell lines increased and that the elevated expression of miR-331-3p promotes proliferation of SMMC7721 cells by inhibiting the target gene.^[22]^ Previous study has identified that Exo-miR-1225-5p was highly expressed in gastric malignant ascite samples, and miR-1225-5p was confirmed to be associated with serosal invasion in gastric cancer.^[23]^ An extensive S-Exo-miRNA profiling of adenocarcinoma and squamous cell carcinoma (SCC) patients paired with healthy donors was performed using miRNA-Seq, showing that miR-320b was upregulated in SCC samples and miR-320b was SCC-specific.^[24]^ These findings were not fully consistent with the literature, indicating that the secretion of exosomal miRNAs is not random but has a strict sorting mechanism, suggesting a specific selective mechanism of release of exosomal miRNAs by cancer cells to regulate cancer progression.^[14]^ Exosomes derived from miR-140-5p overexpressing synovial mesenchymal stem cells (SMSC-140 s) may be effective in treating osteoarthritis.^[25]^ In this process, the transfer of Exo-miRNAs to a recipient cell where they can regulate target gene expression is of particular interest, both in understanding the basic biology of cancer progression and for the development of therapeutic approaches.^[16]^ Moreover, the exosomes could encapsulate and protect miRNAs, cross the biological barrier, and transport them to target cells to function. Therefore, it is far reaching to study the DE-S-Exo-miRNAs and the prediction of potential targets. We believe that this cancer treatment principle is still applicable.

Regarding the results of the PPI analysis, 10 genes with a high degree score were obtained. SMAD4 was identified in the first whole-exome sequencing study of PC, as a tumor suppressor gene.^[43]^ Actin-related protein 2/3 (ARP2/3) complex is an actin nucleator responsible for actin cytoskeleton branching, which is essential for efficient cell migration.^[44]^ A recent study revealed that the loss of SH3GL2 promotes the migration and invasion behaviors of glioblastoma cells.^[45]^ TGN38/41 is an integral membrane protein predominantly located in the trans Golgi network (TGN) of the rat cells.^[46]^ Studies have demonstrated that blocking Cdk2 kinase activity could inhibit the proliferation of human pancreatic cancer cell lines with various genetic alterations, such as mutations in the K-ras, p53, and p16 genes.^[47]^ MRTO4 is one of the trans-acting factors involved in ribosome biogenesis, which in higher eukaryotic cells contains a C-terminal extension similar to the C-terminal part of ribosomal P proteins.^[48]^ A recent genome-wide pathway analysis conducted in PC shows that ADCY9 may play a fundamental role in situations where the fine interplay between intracellular calcium and cAMP determines the cellular function and may be a physiologically relevant docking site for calcineurin.^[49]^

Module analysis of the PPI network demonstrated that the PC process was associated with the TGF-β signaling pathway via the activin A receptor type 2A (ACVR2A), activin A receptor type 2B (ACVR2B) and SMAD4. In the process of TGF-βinduced EMT, Smad complexes can cooperate with the transcription pathways in the control of gene expression in EMT.^[50,51]^ With respect to the role of SMAD4 loss in tumorigenesis, studies have reported that SMAD4 loss relaxes the antiproliferative effects of the TGF-β signaling pathway.^[52]^ Activins are members of the TGF-βfamily of ligands that have multiple biological functions as serum levels of activin A were found to be elevated in pathological conditions, such as cachexia, osteoporosis and cancer.^[53]^ Activin A inhibits BMP-signaling by binding ACVR2A and ACVR2B to promote tumorigenesis.^[54]^ These findings were consisted with our results, indicating that the TGF-βsignaling pathway and related genes were promising candidates for therapeutic intervention.

As for the previous results regarding the regulatory network of 9 main DE-S-Exo-miRNAs and their targets, 5 were considered the most connective target genes among the 9 DE-S-Exo-miRNAs. Moreover, 3 apoptosis genes and their 4 anticorrelated miRNAs were identified. Previous study showed that SH3TC2 is one of the most prevalent genes across the tumor tissue. Strong correlations with the methylation level are more likely to be associated with cancer outcomes and decreased survival rates in multiple tumor types, including breast invasive carcinoma, colon adenocarcinoma, bladder urothelial carcinoma, and cervical squamous cell carcinoma.^[55]^ Emerging evidence has demonstrated that abnormal GRIA2 expression has also been demonstrated in several tumors and tumor cell lines and is thought to increase cell proliferation.^[56,57]^ The expression the SNP rs9390123 of PHACTR2 would significantly increase the risk of lung cancer especially for smokers, but it's functional mechanism is unclear.^[58]^ It has been postulated that, PHACTR2 may have a crucial role in the DNA repair capacity.^[59]^ NTS could stimulate the proliferation of many non-neoplastic tissues as well as the tumorigenesis of multiple tumors.^[60,61]^ A previous study has shown that NTS could activate the pancreatic proliferation through increasing the pancreatic weight, DNA, RNA and protein contents as well as the lipase and amylase concentrations.^[62]^ CDH2 belongs to the cadherin family, is one of the most important biomarkers of EMT, and is significant in the invasion and metastases of tumors.^[63]^ NTS and PHACTR2 were predicted as target genes of exosomal-miR-125a-3p.

Interactions between apoptosis and miR-630,^[64]^ miR-125a-3p,^[65]^ miR-490-5p,^[66]^ and miR-320b^[67]^ were observed to be experimentally validated interactions. The relationship between miR-630 and NTS, CDH2 and GRIA2, miR-490-5p and CDH2 and GRIA2, miR-125a-3p and NTS, miR-320b and CDH2 in the apoptosis process of tumors need future experimental verification. PI3K/Akt/mTOR pathway is widely distributed in cells and is recognized as an important signaling pathway involved in diverse metabolisms, such as cell growth, proliferation and apoptosis.^[68]^ It is reported that tumor cell proliferation can be inhibited by blocking the PI3K/Akt/mTOR pathway during the use of some anti-cancer drugs.^[69]^ Inhibiting the expression of PI3K could enhance cell autophagy and apoptosis by regulating the PI3K/Akt/mTOR pathway.^[70,71]^ MicroRNAs have been implicated in many critical cellular processes including apoptosis. Moreover, miR-630 maintains the apoptotic balance by targeting multiple modulators, such as poly (ADP-ribose) polymerase family member 3 (PARP3), DNA damage inducible transcript 4 (DDIT4), E1A binding protein p300 (EP300) and EP300 downstream effector p53.^[64]^ Another finding suggested that the over-expression of miR-630 also induced apoptosis in pancreatic cancer cells and inhibited target protein insulin-like growth factor 1 receptor (IGF-1R) mRNA and protein expression.^[72]^ According to the results of the present study, it was hypothesized that miR-125a-3p and miR-630 may affect the expression of its target, NTS, CDH2, and GRIA2, through the PI3K/Akt pathway and thus regulate the process of apoptosis, resulting in the inhibition of PC.

This is the first study to use bioinformatics analysis to investigate the serum exosomal miRNAs involved in the carcinogenesis and progression of PC compared with control samples. Tumor exosome miRNA uptake affected the premetastatic organ stroma cells, supporting tumor cell hosting.^[73]^ Compared with previous studies, the present study found several novel serum exosomal-miRNAs as well as several novel genes, including SH3TC2, GRIA2, PHACTR2, NTS, and CDH2, which yielded clues indicating that there are 3 target genes, NTS, CDH2, and GRIA2, that were highly correlated with the apoptosis process of PC. These genes may be potential therapeutic targets. We also discovered potential interactions between these target genes, and if the function of these genes in PC was verified, the genes could potentially be utilized as markers in the early diagnosis or treatment target of pancreatic carcinoma. This work also indicated that cancer-derived exosomal miRNAs are able to target specific recipient cells, as well as promote or inhibit the development of metastatic disease. Therefore, a comprehensive bioinformatic analysis of DE-S-Exo-miRNAs and their targets was necessary. This analysis could provide promising candidate targets for early diagnosis and therapeutic intervention.

There are several limitations of this study. First, we used a small set of samples for the serum exosomal miRNA expression profiling. Thus, further studies with larger sample sizes are warranted. Second, our conclusions are based solely on the results of computational analysis and the same miRNAs may have a tumor suppressive or oncogenic effect depending upon the cell type in which they are expressed. Therefore, future investigations aim to perform some deep experiments to confirm the miRNAs and the predicted miRNA targets in the present study were expected.

Our study provides a comprehensive bioinformatic analysis of DE-S-Exo-miRNAs and their targets, which may be involved in the tumorigenicity of PC. The GO term, KEGG pathway analysis, and PPI network analysis provided a series of related key genes and pathways that contribute to the understanding of the molecular mechanisms of PC and the serum exosomal miRNAs may serve as potential biomarkers for early PC detection. Furthermore, these predictions need further experimental validation in future studies.

## Author contributions

**Conceptualization:** Yiming Ren.

**Data curation:** Xin Zhao.

**Formal analysis:** Xin Zhao, Yiming Ren.

**Investigation:** Naiqiang Cui.

**Methodology:** Xin Zhao, Yiming Ren, Ximo Wang.

**Project administration:** Yunfeng Cui.

**Resources:** Xin Zhao.

**Software:** Xin Zhao, Yiming Ren.

**Supervision:** Naiqiang Cui, Ximo Wang, Yunfeng Cui.

**Writing – original draft:** Xin Zhao.

**Writing – review & editing:** Xin Zhao.

## Supplementary Material

Supplemental Digital Content
