# Supplementary material for: Identification of key microRNAs and their targets in exosomes of pancreatic cancer using bioinformatics analysis
Source: Medicine (Baltimore). 2018 Sep 28;97(39):e12632. doi: 10.1097/MD.0000000000012632 (PMC6181532; doi:10.1097/MD.0000000000012632)
Supplement: Supplemental Digital Content [file medi-97-e12632-s001.doc]

Supplementary materials 1: List of differentially expressed exosomal miRNAs.

| miRNA | adjusted .P.Value | P.Value | Calculated value of t statistic | logFC |
| --- | --- | --- | --- | --- |
| Down-regulated miRNAs |  |  |  |  |
| hsa-miR-3200-3p | 0.0046999 | 1.02E-03 | -28.821 | -10.8135 |
| hsa-miR-595 | 0.0079029 | 2.11E-03 | -20.257 | -7.1454 |
| hsa-miR-668 | 0.0195609 | 6.68E-03 | -11.52 | -7.1363 |
| hsa-miR-491-3p | 0.0211661 | 7.30E-03 | -11.032 | -7.1345 |
| hsa-miR-1909-5p | 0.0090451 | 2.51E-03 | -18.598 | -6.145 |
| hsa-miR-3150b-3p | 0.035254 | 1.30E-02 | -8.31 | -5.5872 |
| hsa-miR-671-5p | 0.0120355 | 3.65E-03 | -15.494 | -3.441 |
| Up-regulated miRNAs |  |  |  |  |
| hsa-miR-3195 | 0.0000302 | 2.36E-08 | 5122.182 | 15.9997 |
| hsa-miR-144-5p | 0.0050589 | 1.14E-03 | 27.286 | 15.0845 |
| hsa-miR-3692-5p | 0.0008696 | 3.98E-05 | 139.211 | 14.9001 |
| hsa-miR-3945 | 0.0000543 | 3.10E-07 | 1468.052 | 14.4144 |
| hsa-miR-516a-5p | 0.0010928 | 6.42E-05 | 110.38 | 14.396 |
| hsa-miR-149-3p | 0.001016 | 5.35E-05 | 120.631 | 14.3021 |
| hsa-miR-1471 | 0.0003269 | 6.34E-06 | 339.683 | 14.1149 |
| hsa-miR-513a-5p | 0.0010098 | 5.11E-05 | 123.382 | 13.8854 |
| hsa-miR-374a-5p | 0.0040011 | 8.37E-04 | 31.734 | 13.8464 |
| hsa-miR-1254 | 0.0001163 | 1.00E-06 | 830.96 | 13.4958 |
| hsa-miR-198 | 0.0000451 | 1.03E-07 | 2503.65 | 13.374 |
| hsa-miR-1305 | 0.0008075 | 3.12E-05 | 156.687 | 13.1999 |
| hsa-miR-181a-5p | 0.0015829 | 1.49E-04 | 73.405 | 13.1053 |
| hsa-miR-1226-5p | 0.001286 | 9.17E-05 | 92.849 | 13.0598 |
| hsa-miR-339-3p | 0.0005593 | 1.75E-05 | 207.284 | 12.8729 |
| hiv1-miR-H1 | 0.0008075 | 3.45E-05 | 149.22 | 12.7814 |
| hsa-miR-1321 | 0.001213 | 8.13E-05 | 98.453 | 12.7791 |
| hsa-miR-1181 | 0.0004785 | 1.37E-05 | 233.743 | 12.7227 |
| hsa-miR-30b-3p | 0.0010645 | 6.15E-05 | 112.744 | 12.6682 |
| hsa-miR-557 | 0.0012785 | 9.02E-05 | 93.624 | 12.668 |
| hsa-miR-96-5p | 0.0026903 | 4.42E-04 | 43.297 | 12.5647 |
| hsa-miR-887 | 0.0002998 | 4.23E-06 | 413.319 | 12.5548 |
| hsa-miR-374b-5p | 0.0026812 | 4.35E-04 | 43.613 | 12.4983 |
| hsa-miR-660-5p | 0.0033698 | 6.47E-04 | 35.962 | 12.3134 |
| hsa-miR-542-5p | 0.0003188 | 5.00E-06 | 381.211 | 12.3027 |
| hsa-miR-3181 | 0.0013168 | 1.05E-04 | 86.834 | 12.2832 |
| hsa-miR-769-3p | 0.0019749 | 2.37E-04 | 58.534 | 12.2781 |
| hsa-miR-3925-5p | 0.0008075 | 3.39E-05 | 150.627 | 12.2711 |
| hsa-miR-18a-5p | 0.0038358 | 7.87E-04 | 32.704 | 12.2551 |
| hsa-miR-186-5p | 0.003261 | 6.11E-04 | 36.982 | 12.239 |
| hsa-miR-10b-3p | 0.0023977 | 3.44E-04 | 48.886 | 12.222 |
| hsa-miR-125a-3p | 0.003569 | 6.99E-04 | 34.63 | 12.1706 |
| hsa-miR-301a-3p | 0.0035471 | 6.89E-04 | 34.87 | 12.1619 |
| hsa-miR-3713 | 0.0028271 | 4.94E-04 | 40.996 | 12.1219 |
| hsa-miR-4304 | 0.0037161 | 7.46E-04 | 33.553 | 12.0861 |
| hsa-miR-331-3p | 0.0013312 | 1.09E-04 | 85.532 | 12.0247 |
| hsa-miR-195-5p | 0.0037161 | 7.43E-04 | 33.627 | 12.0209 |
| hsa-miR-30c-1-3p | 0.0002913 | 3.65E-06 | 443.767 | 11.9794 |
| hsa-miR-371a-3p | 0.0003291 | 6.71E-06 | 330.476 | 11.9779 |
| hsa-miR-4299 | 0.0272601 | 9.75E-03 | 9.564 | 11.9055 |
| hsa-miR-590-5p | 0.0035141 | 6.80E-04 | 35.097 | 11.8662 |
| hsa-miR-1273e | 0.0008075 | 3.48E-05 | 148.61 | 11.8648 |
| hsa-miR-454-3p | 0.0038358 | 7.84E-04 | 32.76 | 11.819 |
| hsa-miR-3659 | 0.0022054 | 2.94E-04 | 52.766 | 11.7679 |
| hsa-miR-3188 | 0.0000455 | 2.14E-07 | 1759.188 | 11.7618 |
| hsa-miR-140-5p | 0.0052059 | 1.20E-03 | 26.64 | 11.688 |
| hsa-miR-18b-5p | 0.0061411 | 1.48E-03 | 24.032 | 11.6199 |
| hsv2-miR-H21 | 0.0082656 | 2.24E-03 | 19.651 | 11.5338 |
| hsa-miR-663a | 0.0027776 | 4.78E-04 | 41.646 | 11.426 |
| hsa-miR-126-5p | 0.0012017 | 7.91E-05 | 99.766 | 11.3799 |
| hsa-miR-3622b-5p | 0.0032083 | 5.96E-04 | 37.429 | 11.3571 |
| hsa-miR-7-5p | 0.0050953 | 1.16E-03 | 27.054 | 11.3559 |
| hsa-miR-424-5p | 0.0004718 | 1.22E-05 | 247.169 | 11.349 |
| hsa-miR-423-5p | 0.0022813 | 3.19E-04 | 50.708 | 11.3208 |
| hsa-miR-1288 | 0.0004505 | 1.10E-05 | 259.818 | 11.3147 |
| hsa-miR-183-5p | 0.0024049 | 3.47E-04 | 48.686 | 11.3081 |
| hsa-miR-215 | 0.0049408 | 1.10E-03 | 27.837 | 11.2656 |
| hsa-miR-210 | 0.0041862 | 8.79E-04 | 30.983 | 11.2417 |
| hsa-miR-130b-3p | 0.0048977 | 1.08E-03 | 28.052 | 11.2175 |
| hsa-miR-3655 | 0.0013017 | 1.03E-04 | 87.759 | 11.1629 |
| hsa-miR-148b-3p | 0.0016235 | 1.62E-04 | 70.517 | 11.1393 |
| hsa-miR-199a-5p | 0.0023977 | 3.42E-04 | 48.991 | 11.1123 |
| hsa-miR-3137 | 0.0013816 | 1.17E-04 | 82.529 | 11.0075 |
| hsa-miR-345-5p | 0.002765 | 4.70E-04 | 41.993 | 10.9985 |
| hbv-miR-B20 | 0.0033243 | 6.30E-04 | 36.417 | 10.9917 |
| hsa-miR-296-5p | 0.001909 | 2.15E-04 | 61.346 | 10.9895 |
| hsa-miR-4294 | 0.0020368 | 2.53E-04 | 56.762 | 10.9207 |
| kshv-miR-K12-10b | 0.0001432 | 1.57E-06 | 668.429 | 10.8129 |
| hsa-miR-151a-3p | 0.0003203 | 5.52E-06 | 363.129 | 10.7832 |
| hsa-miR-221-3p | 0.004851 | 1.06E-03 | 28.232 | 10.7474 |
| hsa-miR-548q | 0.0010098 | 5.16E-05 | 122.727 | 10.7375 |
| hsa-miR-3180-3p | 0.0036058 | 7.12E-04 | 34.325 | 10.7352 |
| hsa-miR-3154 | 0.0011426 | 7.34E-05 | 103.443 | 10.6759 |
| hsa-miR-320c | 0.0059528 | 1.43E-03 | 24.477 | 10.6132 |
| hsa-miR-520b | 0.0018764 | 2.01E-04 | 63.375 | 10.5734 |
| hsa-miR-16-2-3p | 0.0071973 | 1.83E-03 | 21.704 | 10.5162 |
| hsv1-miR-H15 | 0.0003188 | 4.82E-06 | 387.976 | 10.4687 |
| hsa-miR-3196 | 0.0012222 | 8.33E-05 | 97.279 | 10.4325 |
| hsa-miR-892b | 0.0011426 | 7.25E-05 | 104.062 | 10.4291 |
| hsa-miR-373-5p | 0.0012761 | 8.90E-05 | 94.218 | 10.393 |
| hsa-miR-659-3p | 0.0050793 | 1.15E-03 | 27.141 | 10.3849 |
| hsa-miR-4274 | 0.0018909 | 2.07E-04 | 62.502 | 10.3692 |
| hsa-miR-936 | 0.0007815 | 2.94E-05 | 161.407 | 10.326 |
| hsa-miR-338-3p | 0.0402588 | 1.51E-02 | 7.707 | 10.2888 |
| hsa-miR-490-5p | 0.001016 | 5.43E-05 | 119.732 | 10.2812 |
| hsv2-miR-H2 | 0.002549 | 4.02E-04 | 45.303 | 10.2613 |
| hsa-miR-532-5p | 0.0047649 | 1.04E-03 | 28.579 | 10.1789 |
| hsa-miR-146b-5p | 0.0025065 | 3.81E-04 | 46.506 | 10.166 |
| hsa-miR-187-5p | 0.0008075 | 3.35E-05 | 151.432 | 10.1406 |
| hsa-miR-500a-5p | 0.0016235 | 1.63E-04 | 70.212 | 10.1307 |
| hsa-miR-422a | 0.0003269 | 6.38E-06 | 338.642 | 10.0547 |
| hsa-miR-128 | 0.0033698 | 6.47E-04 | 35.967 | 10.0461 |
| hsa-miR-17-3p | 0.0091606 | 2.56E-03 | 18.418 | 10.0357 |
| hsa-miR-1295a | 0.0002279 | 2.68E-06 | 515.84 | 10.0208 |
| hsa-miR-197-3p | 0.0018788 | 2.03E-04 | 63.113 | 9.9544 |
| hsa-miR-340-5p | 0.0018909 | 2.09E-04 | 62.263 | 9.9482 |
| hsa-miR-139-3p | 0.0003188 | 5.25E-06 | 372.287 | 9.9175 |
| hsa-miR-202-3p | 0.0022433 | 3.04E-04 | 51.888 | 9.909 |
| hsa-miR-299-3p | 0.0002998 | 4.02E-06 | 423.707 | 9.8562 |
| hsa-miR-361-5p | 0.0018379 | 1.93E-04 | 64.709 | 9.8394 |
| hsa-miR-376a-3p | 0.0027655 | 4.72E-04 | 41.896 | 9.8195 |
| hsa-miR-10b-5p | 0.0402588 | 1.51E-02 | 7.713 | 9.8187 |
| hsa-miR-423-3p | 0.0059868 | 1.44E-03 | 24.37 | 9.7715 |
| hsa-miR-575 | 0.0024347 | 3.55E-04 | 48.142 | 9.7661 |
| hsa-let-7e-5p | 0.0074004 | 1.90E-03 | 21.285 | 9.7602 |
| hsa-miR-3676-3p | 0.0020368 | 2.52E-04 | 56.818 | 9.7106 |
| hsa-miR-324-5p | 0.0010645 | 6.17E-05 | 112.526 | 9.7059 |
| hsa-miR-3621 | 0.0016235 | 1.62E-04 | 70.392 | 9.6838 |
| hsa-miR-3200-5p | 0.0004785 | 1.38E-05 | 233.009 | 9.6636 |
| hsv2-miR-H9-5p | 0.0004815 | 1.43E-05 | 228.531 | 9.6287 |
| hsa-miR-509-3-5p | 0.0007815 | 2.94E-05 | 161.298 | 9.6274 |
| hsa-miR-520e | 0.0025173 | 3.89E-04 | 46.034 | 9.568 |
| hsa-miR-3938 | 0.0044738 | 9.54E-04 | 29.783 | 9.5657 |
| hsa-miR-362-5p | 0.0074069 | 1.91E-03 | 21.244 | 9.5393 |
| hsa-miR-1273c | 0.0011027 | 6.65E-05 | 108.504 | 9.5375 |
| hsa-miR-501-5p | 0.0004505 | 1.13E-05 | 256.564 | 9.529 |
| hsa-miR-665 | 0.0016039 | 1.57E-04 | 71.504 | 9.5199 |
| hsa-miR-3164 | 0.0016603 | 1.72E-04 | 68.479 | 9.5123 |
| hsa-miR-138-5p | 0.0027776 | 4.83E-04 | 41.439 | 9.4899 |
| hsa-miR-492 | 0.0217281 | 7.56E-03 | 10.843 | 9.4578 |
| hsa-miR-711 | 0.0026467 | 4.23E-04 | 44.201 | 9.4221 |
| hsa-miR-519e-5p | 0.0027776 | 4.82E-04 | 41.488 | 9.417 |
| hsa-miR-516a-3p | 0.0012248 | 8.45E-05 | 96.644 | 9.3768 |
| hsa-miR-125a-5p | 0.0095176 | 2.69E-03 | 17.969 | 9.3765 |
| hsa-miR-625-5p | 0.0014978 | 1.35E-04 | 76.974 | 9.3627 |
| hsa-miR-28-5p | 0.0044124 | 9.34E-04 | 30.091 | 9.3526 |
| hcmv-miR-US5-1 | 0.0003294 | 6.97E-06 | 324.345 | 9.3473 |
| hsa-miR-3666 | 0.0006635 | 2.34E-05 | 180.198 | 9.3375 |
| hsa-miR-1469 | 0.0027416 | 4.62E-04 | 42.357 | 9.3255 |
| hsv2-miR-H3 | 0.001213 | 8.18E-05 | 98.189 | 9.3158 |
| hsa-miR-30e-3p | 0.0096545 | 2.74E-03 | 17.821 | 9.2943 |
| hsa-miR-629-3p | 0.0000451 | 1.77E-07 | 1930.29 | 9.2849 |
| hsa-miR-32-5p | 0.0137287 | 4.28E-03 | 14.327 | 9.2514 |
| hsa-miR-335-5p | 0.0053579 | 1.25E-03 | 26.14 | 9.2042 |
| hsa-miR-365a-3p | 0.0031222 | 5.64E-04 | 38.445 | 9.1846 |
| hsa-let-7d-3p | 0.001909 | 2.15E-04 | 61.401 | 9.1588 |
| hsa-miR-516b-5p | 0.0014751 | 1.31E-04 | 78.208 | 9.108 |
| hsa-miR-182-5p | 0.0026903 | 4.48E-04 | 42.994 | 9.1061 |
| hsa-miR-484 | 0.0024152 | 3.50E-04 | 48.457 | 9.0511 |
| hsa-miR-1208 | 0.0026749 | 4.32E-04 | 43.766 | 9.0339 |
| hsa-miR-378_v17.0 | 0.0016418 | 1.67E-04 | 69.365 | 9.015 |
| hsa-miR-222-3p | 0.0004022 | 8.83E-06 | 289.239 | 8.9928 |
| hsa-miR-574-3p | 0.0013017 | 1.03E-04 | 87.879 | 8.9662 |
| hsa-miR-526b-5p | 0.002765 | 4.70E-04 | 42.019 | 8.9603 |
| hsv1-miR-H14-3p | 0.0019749 | 2.38E-04 | 58.408 | 8.9448 |
| hsa-miR-1275 | 0.001909 | 2.13E-04 | 61.647 | 8.935 |
| hsa-miR-1307-3p | 0.0012872 | 9.42E-05 | 91.681 | 8.9051 |
| hsa-miR-3177-3p | 0.0000451 | 1.42E-07 | 2147.363 | 8.8933 |
| hsa-miR-550a-3p | 0.0138731 | 4.34E-03 | 14.237 | 8.8926 |
| hsa-miR-2861 | 0.0019479 | 2.27E-04 | 59.867 | 8.8912 |
| hsa-miR-3675-3p | 0.0022813 | 3.21E-04 | 50.517 | 8.8652 |
| hsa-miR-370 | 0.0003269 | 6.40E-06 | 337.927 | 8.8624 |
| hsa-miR-3663-3p | 0.0013017 | 1.03E-04 | 87.826 | 8.858 |
| hsv2-miR-H5 | 0.0024431 | 3.62E-04 | 47.69 | 8.853 |
| hsa-miR-520c-3p | 0.0013454 | 1.12E-04 | 84.362 | 8.8516 |
| hsa-miR-29c-5p | 0.0026903 | 4.47E-04 | 43.059 | 8.8507 |
| hsa-miR-30c-2-3p | 0.0026247 | 4.18E-04 | 44.486 | 8.8033 |
| hsa-miR-638 | 0.0010645 | 5.92E-05 | 114.804 | 8.7986 |
| hsa-miR-3163 | 0.0022038 | 2.91E-04 | 52.983 | 8.7589 |
| hsa-miR-342-5p | 0.001016 | 5.49E-05 | 119.081 | 8.7519 |
| hsa-miR-10a-5p | 0.0401644 | 1.50E-02 | 7.736 | 8.7386 |
| hsa-miR-125b-5p | 0.0025173 | 3.89E-04 | 46.066 | 8.7373 |
| hsa-miR-361-3p | 0.0008075 | 3.22E-05 | 154.383 | 8.718 |
| hsa-miR-184 | 0.0026903 | 4.43E-04 | 43.226 | 8.7114 |
| hsa-miR-3679-3p | 0.0046999 | 1.02E-03 | 28.851 | 8.7061 |
| hsa-miR-625-3p | 0.0026531 | 4.26E-04 | 44.044 | 8.7038 |
| hsa-miR-508-5p | 0.0022296 | 2.99E-04 | 52.304 | 8.6949 |
| hsa-miR-532-3p | 0.0107047 | 3.13E-03 | 16.7 | 8.6946 |
| hsa-miR-100-5p | 0.000582 | 1.96E-05 | 196.344 | 8.6927 |
| hsa-miR-15b-5p | 0.0063616 | 1.57E-03 | 23.403 | 8.6916 |
| hsa-miR-4251 | 0.001125 | 6.97E-05 | 106.125 | 8.6876 |
| hsa-miR-1260b | 0.0014978 | 1.34E-04 | 77.196 | 8.6779 |
| hsa-miR-362-3p | 0.0115666 | 3.46E-03 | 15.894 | 8.6641 |
| hsa-miR-658 | 0.0016039 | 1.56E-04 | 71.767 | 8.6507 |
| hsa-miR-3190-3p | 0.0010098 | 5.04E-05 | 124.17 | 8.6445 |
| hsa-miR-486-3p | 0.0053835 | 1.26E-03 | 26.037 | 8.621 |
| hsa-miR-3180 | 0.0011241 | 6.87E-05 | 106.828 | 8.6075 |
| hsa-miR-513b | 0.0000543 | 3.40E-07 | 1403.768 | 8.6051 |
| hsa-miR-20a-3p | 0.0134417 | 4.17E-03 | 14.512 | 8.6016 |
| hsa-miR-564 | 0.0063229 | 1.54E-03 | 23.582 | 8.5805 |
| hsa-miR-298 | 0.0016601 | 1.70E-04 | 68.736 | 8.5641 |
| hsa-miR-1323 | 0.0015242 | 1.39E-04 | 76.003 | 8.5588 |
| hsa-miR-377-3p | 0.0068133 | 1.70E-03 | 22.461 | 8.5586 |
| hsa-miR-500a-3p | 0.0163164 | 5.40E-03 | 12.794 | 8.5477 |
| hsa-miR-627 | 0.0074728 | 1.93E-03 | 21.121 | 8.5128 |
| hsa-let-7f-1-3p | 0.0016005 | 1.52E-04 | 72.621 | 8.4926 |
| hsa-let-7b-3p | 0.0018909 | 2.08E-04 | 62.37 | 8.4853 |
| hsa-miR-3680-3p | 0.0024431 | 3.61E-04 | 47.774 | 8.4689 |
| hsa-miR-744-5p | 0.0019749 | 2.37E-04 | 58.622 | 8.4647 |
| hsa-miR-766-3p | 0.0004505 | 1.09E-05 | 261.278 | 8.4625 |
| hsa-miR-365b-5p | 0.0011027 | 6.57E-05 | 109.146 | 8.4523 |
| hsa-miR-196b-5p | 0.0098149 | 2.81E-03 | 17.607 | 8.4385 |
| hsa-miR-4318 | 0.0072807 | 1.86E-03 | 21.526 | 8.4363 |
| hsa-miR-3185 | 0.0016039 | 1.57E-04 | 71.571 | 8.418 |
| hsa-miR-3183 | 0.001286 | 9.27E-05 | 92.369 | 8.4135 |
| hsa-miR-3138 | 0.0044421 | 9.43E-04 | 29.939 | 8.4124 |
| hsa-miR-3926 | 0.0000773 | 5.45E-07 | 1116.79 | 8.4106 |
| hsa-miR-3667-5p | 0.0037161 | 7.48E-04 | 33.505 | 8.4073 |
| hsa-miR-190a | 0.0090778 | 2.53E-03 | 18.54 | 8.4026 |
| hsa-miR-502-3p | 0.0111051 | 3.29E-03 | 16.297 | 8.4017 |
| hsa-miR-3065-3p | 0.0008697 | 4.09E-05 | 137.427 | 8.3848 |
| hsa-miR-374c-5p | 0.0065188 | 1.61E-03 | 23.055 | 8.3667 |
| hsa-miR-136-5p | 0.0012872 | 9.48E-05 | 91.367 | 8.3625 |
| hsa-miR-518a-5p | 0.0035924 | 7.07E-04 | 34.453 | 8.3563 |
| hsa-miR-1306-3p | 0.0032083 | 5.96E-04 | 37.432 | 8.3515 |
| hsa-miR-708-5p | 0.0073844 | 1.89E-03 | 21.339 | 8.35 |
| hsa-miR-518c-5p | 0.0025449 | 3.99E-04 | 45.487 | 8.3381 |
| hsa-miR-636 | 0.0016005 | 1.53E-04 | 72.428 | 8.3208 |
| hsa-miR-486-5p | 0.0194906 | 6.63E-03 | 11.564 | 8.3203 |
| hsa-miR-20b-5p | 0.0082834 | 2.26E-03 | 19.575 | 8.3097 |
| hsa-miR-10a-3p | 0.0022038 | 2.87E-04 | 53.363 | 8.3032 |
| hsa-miR-3654 | 0.0024514 | 3.67E-04 | 47.38 | 8.2896 |
| hsa-miR-149-5p | 0.0019214 | 2.18E-04 | 60.948 | 8.2892 |
| hsa-miR-18b-3p | 0.0036789 | 7.29E-04 | 33.927 | 8.2888 |
| hsa-miR-4292 | 0.00083 | 3.64E-05 | 145.365 | 8.2854 |
| hsa-miR-3197 | 0.0007815 | 2.91E-05 | 162.051 | 8.2685 |
| hsa-miR-563 | 0.0014656 | 1.29E-04 | 78.793 | 8.2671 |
| hsa-miR-487b | 0.0049806 | 1.11E-03 | 27.634 | 8.2402 |
| hsa-miR-513c-5p | 0.0001432 | 1.47E-06 | 690.648 | 8.2376 |
| hsa-miR-125b-2-3p | 0.0038358 | 7.84E-04 | 32.755 | 8.2304 |
| hsa-miR-409-3p | 0.0022659 | 3.14E-04 | 51.067 | 8.2284 |
| hsa-miR-3928 | 0.0008696 | 4.02E-05 | 138.558 | 8.2188 |
| hsa-miR-4284 | 0.0022813 | 3.22E-04 | 50.485 | 8.1752 |
| hsa-miR-22-5p | 0.000582 | 1.96E-05 | 196.309 | 8.1732 |
| hsa-miR-3180-5p | 0.0024431 | 3.62E-04 | 47.689 | 8.1605 |
| hsa-miR-16-5p | 0.006342 | 1.56E-03 | 23.474 | 8.156 |
| hsa-miR-133b | 0.0004785 | 1.39E-05 | 232.213 | 8.1536 |
| hsa-miR-3622a-5p | 0.0020368 | 2.54E-04 | 56.654 | 8.1526 |
| hsa-miR-3161 | 0.0071973 | 1.83E-03 | 21.708 | 8.1336 |
| hsa-miR-3923 | 0.006811 | 1.70E-03 | 22.499 | 8.108 |
| hsa-miR-181b-5p | 0.0014317 | 1.23E-04 | 80.395 | 8.1009 |
| hsa-miR-513a-3p | 0.0009212 | 4.48E-05 | 131.53 | 8.0979 |
| hsa-let-7d-5p | 0.0052059 | 1.20E-03 | 26.623 | 8.065 |
| hsa-miR-1972 | 0.0013312 | 1.10E-04 | 85.187 | 8.0404 |
| hsa-miR-92a-3p | 0.0308937 | 1.13E-02 | 8.91 | 8.0381 |
| hsa-miR-26b-5p | 0.0071973 | 1.82E-03 | 21.727 | 8.0321 |
| hsa-miR-622 | 0.0029889 | 5.29E-04 | 39.644 | 8.0209 |
| hsa-miR-3130-3p | 0.0015272 | 1.41E-04 | 75.302 | 8.0033 |
| hsa-miR-1303 | 0.0013017 | 9.88E-05 | 89.573 | 8.0021 |
| hsa-miR-302c-5p | 0.0010098 | 5.22E-05 | 122.036 | 7.994 |
| hsa-miR-3907 | 0.0010645 | 6.00E-05 | 114.127 | 7.9847 |
| hsa-miR-107 | 0.006342 | 1.55E-03 | 23.484 | 7.9471 |
| hsa-miR-663b | 0.001351 | 1.13E-04 | 83.808 | 7.9099 |
| hsa-miR-4317 | 0.0116059 | 3.49E-03 | 15.827 | 7.8745 |
| hsa-miR-551b-5p | 0.0150675 | 4.87E-03 | 13.46 | 7.8733 |
| hsa-miR-185-5p | 0.0188762 | 6.37E-03 | 11.799 | 7.8684 |
| hsa-miR-410 | 0.0032083 | 5.90E-04 | 37.623 | 7.866 |
| hsa-miR-20a-5p | 0.0078952 | 2.09E-03 | 20.325 | 7.8553 |
| hsa-miR-4312 | 0.0019479 | 2.27E-04 | 59.749 | 7.8484 |
| hsa-miR-15a-5p | 0.0084087 | 2.31E-03 | 19.378 | 7.8245 |
| hsa-miR-133a | 0.0031085 | 5.55E-04 | 38.73 | 7.8188 |
| hsa-miR-505-3p | 0.0108057 | 3.18E-03 | 16.58 | 7.8048 |
| hsa-miR-25-3p | 0.0131893 | 4.07E-03 | 14.683 | 7.8034 |
| hsa-miR-502-5p | 0.016232 | 5.36E-03 | 12.842 | 7.7829 |
| hsa-miR-1251 | 0.0026916 | 4.51E-04 | 42.834 | 7.7774 |
| hsa-miR-3132 | 0.002139 | 2.77E-04 | 54.336 | 7.7708 |
| hsv2-miR-H20 | 0.0037514 | 7.59E-04 | 33.288 | 7.7617 |
| hsa-miR-99a-5p | 0.0076971 | 2.03E-03 | 20.637 | 7.76 |
| hsa-miR-624-5p | 0.0227832 | 8.00E-03 | 10.546 | 7.7553 |
| hsa-miR-3120-3p | 0.0038358 | 7.90E-04 | 32.634 | 7.7525 |
| hsa-miR-617 | 0.0012017 | 7.90E-05 | 99.809 | 7.7474 |
| hsa-miR-3122 | 0.0019749 | 2.36E-04 | 58.706 | 7.7468 |
| hsa-miR-718 | 0.0020386 | 2.56E-04 | 56.457 | 7.745 |
| hsa-miR-212-3p | 0.0014557 | 1.27E-04 | 79.398 | 7.709 |
| hsa-miR-372 | 0.0024514 | 3.67E-04 | 47.369 | 7.6892 |
| hsa-miR-1470 | 0.0004785 | 1.35E-05 | 235.591 | 7.6486 |
| hsa-miR-629-5p | 0.031179 | 1.14E-02 | 8.86 | 7.6241 |
| hsa-miR-494 | 0.0110874 | 3.28E-03 | 16.331 | 7.6177 |
| hsa-let-7g-5p | 0.0056033 | 1.32E-03 | 25.411 | 7.5897 |
| hsa-miR-3187-3p | 0.0005165 | 1.58E-05 | 218.124 | 7.5786 |
| hsa-miR-19a-3p | 0.014147 | 4.48E-03 | 14.016 | 7.5642 |
| hsa-let-7a-5p | 0.0062488 | 1.51E-03 | 23.792 | 7.5399 |
| hsa-miR-1228-5p | 0.0008696 | 3.96E-05 | 139.522 | 7.53 |
| hsa-miR-339-5p | 0.001567 | 1.46E-04 | 74.064 | 7.5056 |
| hsa-miR-140-3p | 0.0155575 | 5.07E-03 | 13.188 | 7.5022 |
| hsa-miR-515-3p | 0.0004505 | 1.06E-05 | 264.307 | 7.4925 |
| hsa-miR-125b-1-3p | 0.0161491 | 5.32E-03 | 12.889 | 7.4833 |
| hsa-miR-223-5p | 0.0075836 | 1.98E-03 | 20.878 | 7.4816 |
| hsa-miR-1247-5p | 0.0010645 | 6.12E-05 | 113.001 | 7.4681 |
| hsa-miR-150-5p | 0.0019836 | 2.41E-04 | 58.101 | 7.4673 |
| hsa-miR-1180 | 0.0025173 | 3.88E-04 | 46.084 | 7.4585 |
| hsa-miR-518e-5p | 0.0018223 | 1.90E-04 | 65.214 | 7.453 |
| hsa-miR-181c-5p | 0.0120383 | 3.67E-03 | 15.449 | 7.4454 |
| hsa-miR-103a-3p | 0.0052059 | 1.20E-03 | 26.596 | 7.4277 |
| hsa-let-7f-5p | 0.0069512 | 1.74E-03 | 22.209 | 7.4256 |
| hsa-miR-132-3p | 0.0080364 | 2.17E-03 | 19.95 | 7.4233 |
| hsa-miR-4323 | 0.0025173 | 3.91E-04 | 45.952 | 7.4119 |
| hsa-miR-623 | 0.0150675 | 4.86E-03 | 13.468 | 7.4 |
| hsa-miR-186-3p | 0.025141 | 8.89E-03 | 10.014 | 7.3738 |
| hsa-miR-15b-3p | 0.0149906 | 4.82E-03 | 13.526 | 7.3415 |
| hsa-miR-19b-3p | 0.0152864 | 4.95E-03 | 13.348 | 7.3183 |
| hsa-miR-196a-5p | 0.0307675 | 1.11E-02 | 8.957 | 7.3109 |
| hsa-miR-1915-3p | 0.0032083 | 5.94E-04 | 37.473 | 7.3022 |
| hsa-miR-340-3p | 0.0056033 | 1.31E-03 | 25.486 | 7.2951 |
| hsa-miR-17-5p | 0.0077189 | 2.04E-03 | 20.579 | 7.2936 |
| ebv-miR-BHRF1-1 | 0.014433 | 4.63E-03 | 13.796 | 7.2721 |
| hsa-miR-1915-5p | 0.0013168 | 1.06E-04 | 86.441 | 7.2189 |
| hsa-miR-3665 | 0.0038427 | 7.95E-04 | 32.536 | 7.2127 |
| hsa-miR-505-5p | 0.0103145 | 2.97E-03 | 17.14 | 7.1861 |
| hsa-miR-598 | 0.0049966 | 1.12E-03 | 27.544 | 7.1835 |
| hsa-miR-7-1-3p | 0.0022296 | 3.01E-04 | 52.19 | 7.179 |
| hsa-miR-1268a | 0.002139 | 2.76E-04 | 54.366 | 7.177 |
| hsa-let-7i-5p | 0.0110718 | 3.26E-03 | 16.363 | 7.1639 |
| hsa-miR-199b-5p | 0.0188762 | 6.39E-03 | 11.776 | 7.1617 |
| hsa-miR-1 | 0.0115926 | 3.48E-03 | 15.856 | 7.1467 |
| hsa-miR-628-3p | 0.007485 | 1.94E-03 | 21.066 | 7.107 |
| hsv2-miR-H19 | 0.0022038 | 2.92E-04 | 52.936 | 7.0885 |
| hsa-miR-152 | 0.0012905 | 9.61E-05 | 90.786 | 7.0859 |
| hsa-miR-4269 | 0.0120646 | 3.69E-03 | 15.413 | 7.0576 |
| hsa-miR-654-3p | 0.0056417 | 1.34E-03 | 25.286 | 7.0557 |
| hsa-miR-1182 | 0.0024713 | 3.72E-04 | 47.063 | 7.0517 |
| hsa-miR-3656 | 0.0026903 | 4.41E-04 | 43.309 | 7.0432 |
| hsa-miR-545-3p | 0.007949 | 2.14E-03 | 20.113 | 7.0316 |
| hsa-miR-523-3p | 0.018599 | 6.24E-03 | 11.916 | 7.0285 |
| hsa-miR-30b-5p | 0.0076971 | 2.02E-03 | 20.647 | 7.0168 |
| hsa-miR-181d | 0.0124432 | 3.83E-03 | 15.126 | 6.9862 |
| hsa-miR-497-5p | 0.0076827 | 2.01E-03 | 20.716 | 6.9849 |
| hsv2-miR-H7-3p | 0.0158707 | 5.20E-03 | 13.03 | 6.9403 |
| hsa-miR-3158-3p | 0.0136822 | 4.26E-03 | 14.369 | 6.9263 |
| hsa-miR-34a-5p | 0.0038358 | 7.91E-04 | 32.625 | 6.9242 |
| hsa-miR-342-3p | 0.0013017 | 1.03E-04 | 87.898 | 6.8904 |
| hsa-miR-4306 | 0.0232181 | 8.17E-03 | 10.437 | 6.8613 |
| hsa-miR-4281 | 0.0013017 | 9.91E-05 | 89.43 | 6.8603 |
| hsa-miR-4291 | 0.0011331 | 7.10E-05 | 105.113 | 6.8511 |
| hsa-miR-106b-5p | 0.0142252 | 4.53E-03 | 13.943 | 6.8357 |
| hsa-miR-21-3p | 0.0001063 | 8.33E-07 | 909.092 | 6.7859 |
| hsa-miR-551b-3p | 0.0260695 | 9.26E-03 | 9.815 | 6.7802 |
| hsa-miR-29a-3p | 0.0087265 | 2.40E-03 | 19.004 | 6.7784 |
| hsa-miR-431-5p | 0.0019749 | 2.35E-04 | 58.775 | 6.7665 |
| hsa-miR-1207-5p | 0.0052059 | 1.20E-03 | 26.647 | 6.7327 |
| hsa-miR-630 | 0.0046868 | 1.01E-03 | 29.013 | 6.6913 |
| hsa-miR-130a-3p | 0.0107239 | 3.14E-03 | 16.663 | 6.6824 |
| hsa-miR-320e | 0.0107047 | 3.12E-03 | 16.729 | 6.6569 |
| hsa-miR-30e-5p | 0.0142089 | 4.51E-03 | 13.969 | 6.6345 |
| hsa-miR-378c | 0.0019479 | 2.26E-04 | 59.928 | 6.6063 |
| hsa-miR-3648 | 0.0023977 | 3.41E-04 | 49.094 | 6.6022 |
| hsa-miR-33a-5p | 0.0031566 | 5.74E-04 | 38.118 | 6.5907 |
| hsa-miR-30c-5p | 0.007485 | 1.95E-03 | 21.043 | 6.5872 |
| hsa-miR-135a-3p | 0.0033327 | 6.35E-04 | 36.3 | 6.5841 |
| hsa-miR-223-3p | 0.03083 | 1.12E-02 | 8.929 | 6.5591 |
| hsa-miR-99b-3p | 0.0446963 | 1.68E-02 | 7.308 | 6.5572 |
| hsa-miR-29c-3p | 0.0059039 | 1.41E-03 | 24.618 | 6.5318 |
| hsa-miR-769-5p | 0.0059039 | 1.41E-03 | 24.615 | 6.5209 |
| hsa-miR-572 | 0.0079291 | 2.12E-03 | 20.19 | 6.5113 |
| hsa-miR-101-5p | 0.0401644 | 1.50E-02 | 7.732 | 6.5032 |
| hsa-let-7c | 0.0056033 | 1.32E-03 | 25.429 | 6.4947 |
| hsa-let-7b-5p | 0.0063006 | 1.53E-03 | 23.66 | 6.4906 |
| hsa-miR-199a-3p | 0.0083607 | 2.29E-03 | 19.459 | 6.4725 |
| hsa-miR-648 | 0.0015272 | 1.40E-04 | 75.575 | 6.4547 |
| hsa-miR-3198 | 0.0050325 | 1.13E-03 | 27.402 | 6.4413 |
| hsa-miR-101-3p | 0.0134417 | 4.17E-03 | 14.515 | 6.4027 |
| hsa-miR-3714 | 0.0022602 | 3.08E-04 | 51.532 | 6.3977 |
| hsv2-miR-H10 | 0.0021097 | 2.68E-04 | 55.191 | 6.3582 |
| hsa-miR-3151 | 0.0006573 | 2.27E-05 | 183.003 | 6.3563 |
| hsa-miR-548am-5p | 0.0256737 | 9.09E-03 | 9.9 | 6.3404 |
| hcmv-miR-UL148D | 0.0272601 | 9.76E-03 | 9.56 | 6.2961 |
| hcmv-miR-US33-5p | 0.0001183 | 1.11E-06 | 790.309 | 6.2689 |
| hsv1-miR-H6-5p | 0.0037161 | 7.44E-04 | 33.612 | 6.262 |
| hsa-miR-27a-3p | 0.0140037 | 4.42E-03 | 14.109 | 6.2255 |
| hsa-miR-628-5p | 0.0359832 | 1.33E-02 | 8.218 | 6.2054 |
| hsa-miR-3141 | 0.0022038 | 2.90E-04 | 53.13 | 6.1603 |
| hsa-miR-150-3p | 0.0042218 | 8.90E-04 | 30.799 | 6.1447 |
| hsa-miR-22-3p | 0.012386 | 3.81E-03 | 15.179 | 6.0931 |
| hsa-miR-301b | 0.0079291 | 2.13E-03 | 20.167 | 6.0862 |
| hsa-miR-194-3p | 0.0025449 | 3.98E-04 | 45.544 | 6.0777 |
| hsa-miR-27a-5p | 0.0008075 | 3.28E-05 | 153.032 | 6.0684 |
| hsa-miR-146a-5p | 0.0026903 | 4.49E-04 | 42.942 | 6.0626 |
| hsa-miR-3131 | 0.0107047 | 3.13E-03 | 16.706 | 6.0214 |
| hsa-miR-3679-5p | 0.0016235 | 1.64E-04 | 70.004 | 5.9478 |
| hsa-miR-24-3p | 0.0078987 | 2.10E-03 | 20.291 | 5.9446 |
| hsa-miR-29b-3p | 0.0005805 | 1.87E-05 | 201.159 | 5.9302 |
| hsa-miR-1183 | 0.0140037 | 4.42E-03 | 14.103 | 5.9278 |
| hsa-miR-1225-5p | 0.0047965 | 1.05E-03 | 28.437 | 5.9242 |
| hsa-miR-642b-3p | 0.0028473 | 5.00E-04 | 40.765 | 5.8372 |
| hsa-miR-181c-3p | 0.0159151 | 5.23E-03 | 12.997 | 5.8257 |
| hsa-miR-15a-3p | 0.0211936 | 7.32E-03 | 11.013 | 5.8238 |
| hsa-miR-1202 | 0.0040011 | 8.37E-04 | 31.728 | 5.8066 |
| hcmv-miR-UL70-3p | 0.0049204 | 1.09E-03 | 27.941 | 5.7773 |
| hsa-miR-501-3p | 0.0139845 | 4.39E-03 | 14.147 | 5.768 |
| hsa-miR-300 | 0.0014213 | 1.21E-04 | 81.039 | 5.7598 |
| hsa-miR-194-5p | 0.0272601 | 9.74E-03 | 9.571 | 5.7532 |
| hsa-miR-21-5p | 0.0018764 | 2.00E-04 | 63.545 | 5.6839 |
| hsa-miR-1224-5p | 0.0071026 | 1.79E-03 | 21.944 | 5.642 |
| hsa-miR-23a-3p | 0.0226446 | 7.93E-03 | 10.59 | 5.6208 |
| hsa-miR-142-3p | 0.0072807 | 1.86E-03 | 21.518 | 5.5802 |
| hsa-miR-566 | 0.0000451 | 1.55E-07 | 2053.474 | 5.4263 |
| hsa-miR-26a-5p | 0.0008964 | 4.29E-05 | 134.337 | 5.4205 |
| ebv-miR-BHRF1-3 | 0.0027776 | 4.80E-04 | 41.567 | 5.4136 |
| hsa-miR-1249 | 0.003476 | 6.70E-04 | 35.353 | 5.405 |
| hsa-miR-126-3p | 0.0046999 | 1.02E-03 | 28.849 | 5.3884 |
| bkv-miR-B1-5p | 0.0115327 | 3.44E-03 | 15.937 | 5.3742 |
| hsa-miR-142-5p | 0.018599 | 6.21E-03 | 11.939 | 5.3552 |
| hsa-miR-4270 | 0.0080221 | 2.16E-03 | 19.995 | 5.3509 |
| hsa-miR-498 | 0.0022602 | 3.10E-04 | 51.389 | 5.3438 |
| hsv2-miR-H22 | 0.0032951 | 6.22E-04 | 36.647 | 5.3301 |
| hsa-miR-3194-5p | 0.0019252 | 2.20E-04 | 60.687 | 5.3075 |
| hsa-miR-4298 | 0.0032951 | 6.22E-04 | 36.669 | 5.2838 |
| hsa-miR-134 | 0.0031222 | 5.65E-04 | 38.402 | 5.28 |
| hsa-miR-30d-5p | 0.0107047 | 3.11E-03 | 16.757 | 5.2766 |
| hsa-miR-144-3p | 0.0386319 | 1.43E-02 | 7.916 | 5.2171 |
| hsa-miR-192-5p | 0.0289154 | 1.04E-02 | 9.276 | 5.1981 |
| hsv1-miR-H17 | 0.0031222 | 5.63E-04 | 38.461 | 5.1602 |
| hsa-miR-583 | 0.0021125 | 2.70E-04 | 54.991 | 5.1324 |
| hsa-miR-320d | 0.0116211 | 3.51E-03 | 15.797 | 5.1198 |
| hsa-miR-27b-3p | 0.0142252 | 4.54E-03 | 13.927 | 5.0692 |
| hsa-miR-188-5p | 0.0120383 | 3.66E-03 | 15.462 | 4.9533 |
| hsa-miR-4314 | 0.0217419 | 7.58E-03 | 10.827 | 4.9377 |
| hsa-miR-23b-3p | 0.0212298 | 7.35E-03 | 10.992 | 4.912 |
| hsa-miR-3917 | 0.0031795 | 5.81E-04 | 37.905 | 4.8434 |
| hsv2-miR-H24 | 0.0032441 | 6.05E-04 | 37.152 | 4.8315 |
| hsa-miR-483-5p | 0.0289373 | 1.04E-02 | 9.262 | 4.8179 |
| hsa-miR-3937 | 0.0067557 | 1.68E-03 | 22.623 | 4.7871 |
| hsa-miR-451a | 0.0018764 | 1.99E-04 | 63.824 | 4.7568 |
| hsa-miR-3156-5p | 0.0350528 | 1.29E-02 | 8.343 | 4.6715 |
| hsa-miR-320b | 0.0089626 | 2.48E-03 | 18.707 | 4.6415 |
| hsa-miR-514b-5p | 0.0453154 | 1.70E-02 | 7.25 | 4.6382 |
| hsa-miR-3127-5p | 0.0094474 | 2.67E-03 | 18.059 | 4.5677 |
| hsa-miR-1225-3p | 0.018599 | 6.23E-03 | 11.923 | 4.5615 |
| hsa-miR-3610 | 0.0050783 | 1.15E-03 | 27.189 | 4.4794 |
| hsv2-miR-H25 | 0.0035566 | 6.94E-04 | 34.756 | 4.4745 |
| hsa-miR-4271 | 0.0044794 | 9.58E-04 | 29.711 | 4.4257 |
| hsa-miR-320a | 0.0052152 | 1.21E-03 | 26.529 | 4.3204 |
| hsa-miR-1228-3p | 0.0049699 | 1.11E-03 | 27.71 | 4.3037 |
| hbv-miR-B2RC | 0.0361291 | 1.33E-02 | 8.193 | 4.2732 |
| hsa-miR-4327 | 0.0056792 | 1.35E-03 | 25.164 | 4.2124 |
| hsa-miR-1914-3p | 0.0021097 | 2.67E-04 | 55.289 | 4.0883 |
| hsa-miR-23a-5p | 0.0194906 | 6.64E-03 | 11.553 | 4.0877 |
| hsa-miR-324-3p | 0.0165859 | 5.51E-03 | 12.663 | 4.0794 |
| hsa-miR-762 | 0.0091661 | 2.57E-03 | 18.376 | 4.0756 |
| hsa-miR-3605-5p | 0.002031 | 2.48E-04 | 57.259 | 4.0685 |
| hsa-miR-933 | 0.0022602 | 3.12E-04 | 51.27 | 3.9715 |
| hsa-miR-601 | 0.0028546 | 5.03E-04 | 40.627 | 3.9353 |
| hsa-miR-4322 | 0.0114392 | 3.41E-03 | 16.021 | 3.8979 |
| hsa-miR-3911 | 0.0122687 | 3.76E-03 | 15.269 | 3.7343 |
| hsa-miR-3202 | 0.0193892 | 6.58E-03 | 11.609 | 3.4689 |
| hsa-miR-2116-3p | 0.0088282 | 2.44E-03 | 18.871 | 3.4253 |
| hsa-miR-3147 | 0.0157867 | 5.16E-03 | 13.079 | 3.3901 |
| hsa-miR-874 | 0.002549 | 4.04E-04 | 45.232 | 3.3703 |
| hsa-miR-940 | 0.0097871 | 2.78E-03 | 17.679 | 3.2531 |
| hsa-miR-371a-5p | 0.0091661 | 2.58E-03 | 18.352 | 3.2227 |
| hsa-miR-193a-5p | 0.0099569 | 2.86E-03 | 17.461 | 3.2171 |
| hsa-miR-602 | 0.0025065 | 3.81E-04 | 46.524 | 3.0174 |
| hsa-miR-378b | 0.0196563 | 6.73E-03 | 11.479 | 3.0147 |
| hsa-miR-634 | 0.0188762 | 6.39E-03 | 11.779 | 2.9405 |
| hsa-miR-155-5p | 0.0105685 | 3.05E-03 | 16.915 | 2.8994 |
| hsa-miR-2276 | 0.015454 | 5.03E-03 | 13.247 | 2.8804 |
| hsa-miR-4253 | 0.0165859 | 5.51E-03 | 12.667 | 2.6806 |
| hcmv-miR-US4 | 0.0293821 | 1.06E-02 | 9.183 | 2.5782 |
| hsa-miR-3125 | 0.0097993 | 2.80E-03 | 17.644 | 2.5391 |
| hsa-miR-191-3p | 0.0091606 | 2.56E-03 | 18.407 | 2.4757 |
| hsa-miR-1299 | 0.0120355 | 3.65E-03 | 15.49 | 2.2883 |
| hsv1-miR-H18 | 0.0390322 | 1.45E-02 | 7.859 | 2.1759 |
| hsa-miR-3614-5p | 0.020642 | 7.10E-03 | 11.182 | 2.1758 |
| hsa-miR-1539 | 0.010607 | 3.07E-03 | 16.863 | 2.1019 |
| hsa-miR-631 | 0.0269927 | 9.60E-03 | 9.638 | 2.0026 |
